# Dysregulated inflammation, oxidative stress, and protein quality control in diabetic HFpEF: unraveling mechanisms and therapeutic targets

**DOI:** 10.1186/s12933-025-02734-4

**Published:** 2025-05-14

**Authors:** Simin Delalat, Innas Sultana, Hersh Osman, Marcel Sieme, Saltanat Zhazykbayeva, Melissa Herwig, Heidi Budde, Árpád Kovács, Mustafa Kaçmaz, Eda Göztepe, Natalie Borgmann, Gelareh Shahriari, Benjamin Sasko, Jan Wintrich, Peter Haldenwang, Wolfgang E. Schmidt, Wiebke Fenske, Muchtiar Khan, Kornelia Jaquet, Andreas Mügge, Domokos Máthé, Viktória E. Tóth, Zoltán V. Varga, Péter Ferdinandy, Ibrahim El-Battrawy, Loek van Heerebeek, Nazha Hamdani

**Affiliations:** 1 Medical Faculty, Department of Cellular and Translational Physiology, Institute of Physiology, Molecular and Experimental Cardiology, Institut Für Forschung und Lehre (IFL), Ruhr University Bochum, 44801 Bochum, Germany; 2 Division of Clinical Physiology, Department of Cardiology, Faculty of Medicine, University of Debrecen, Debrecen, 4032 Hungary; 3 Medical Department II, Marien Hospital Herne, Ruhr University Bochum, Bochum, Germany; 4 Department of Cardiothoracic Surgery, University Hospital Bergmannsheil Bochum, Bochum, Germany; 5 Department of Medicine I, St. Josef Hospital, UK RUB, Ruhr University Bochum, 44801 Bochum, Germany; 6 Department of Internal Medicine I– General Internal Medicine, Endocrinology and Diabetology, Gastroenterology and Hepatology, BG University Hospital Bergmannsheil, Bochum, Germany; 7 Department of Cardiology, OLVG, 1091 AC Amsterdam, The Netherlands; 8 Department of Biophysics and Radiation Biology, Semmelweis University, Tűzoltó utca 37-47, 1094, Budapest, 1085 Hungary; 9 In Vivo Imaging Advanced Core Facility, Hungarian Centre of Excellence for Molecular Medicine, Budapest, Tűzoltó utca 37-47, 1094 Hungary; 10 HCEMM-SU Cardiometabolic Immunology Research Group, Department of Pharmacology and pharmacotherapy,, Semmelweis University, Budapest, 1089 Hungary; 11 Center for Pharmacology and Drug Research & Development,Department of Pharmacology and Pharmacotherapy, Semmelweis University, Budapest, 1089 Hungary; 12 Department of Physiology, Cardiovascular Research Institute, Maastricht University, Maastricht, The Netherlands; 13 Medical Faculty, Department Cellular and Translational Physiology, Institute of Physiology, Ruhr University Bochum, MA 2/156, 44780 Bochum, Germany; 14 Institut Für Forschung und Lehre (IFL), Molecular and Experimental Cardiology, Gudrunstraße 56, 44791 Bochum, Germany; 15 Department of Cardiology, St. Josef-Hospital, UK RUB, Ruhr University Bochum, Bochum, Germany

**Keywords:** Heart failure with preserved ejection fraction, Type 2 diabetes, Inflammation, Oxidative stress, Protein quality control, Insulin resistance, Cardiomyocyte stiffness, Autophagy, Heat shock proteins

## Abstract

**Background:**

Type 2 diabetes mellitus (T2DM) represents a significant risk factor for cardiovascular disease, particularly heart failure with preserved ejection fraction (HFpEF). HFpEF predominantly affects elderly individuals and women, and is characterized by dysfunctions associated with metabolic, inflammatory, and oxidative stress pathways. Despite HFpEF being the most prevalent heart failure phenotype in patients with T2DM, its underlying pathophysiological mechanisms remain inadequately elucidated.

**Objective:**

This study aims to investigate the effects of diabetes mellitus on myocardial inflammation, oxidative stress, and protein quality control (PQC) mechanisms in HFpEF, with particular emphasis on insulin signaling, autophagy, and chaperone-mediated stress responses.

**Methods:**

We conducted an analysis of left ventricular myocardial tissue from HFpEF patients, both with and without diabetes, employing a range of molecular, biochemical, and functional assays. The passive stiffness of cardiomyocytes (Fpassive) was assessed in demembranated cardiomyocytes before and after implementing treatments aimed at reducing inflammation (IL-6 inhibition), oxidative stress (Mito-TEMPO), and enhancing PQC (HSP27, HSP70). Inflammatory markers (NF-κB, IL-6, TNF-α, ICAM-1, VCAM-1, NLRP3), oxidative stress markers (ROS, GSH/GSSG ratio, lipid peroxidation), and components of signaling pathways (PI3K/AKT/mTOR, AMPK, MAPK, and PKG) were evaluated using western blotting, immunofluorescence, and ELISA techniques.

**Results:**

Hearts from diabetic HFpEF patients exhibited significantly heightened inflammation, characterized by the upregulation of NF-κB, IL-6, and the NLRP3 inflammasome. This increase in inflammation was accompanied by elevated oxidative stress, diminished nitric oxide (NO) bioavailability, and impaired activation of the NO-sGC-cGMP-PKG signaling pathway. Notably, dysregulation of insulin signaling was observed, as indicated by decreased AKT phosphorylation and impaired autophagy regulation mediated by AMPK and mTOR. Additionally, PQC dysfunction was evidenced by reduced expression levels of HSP27 and HSP70, which correlated with increased cardiomyocyte passive stiffness. Targeted therapeutic interventions effectively reduced Fpassive, with IL-6 inhibition, Mito-TEMPO, and HSP administration leading to improvements in cardiomyocyte mechanical properties.

**Conclusion:**

The findings of this study elucidate a mechanistic relationship among diabetes, inflammation, oxidative stress, and PQC impairment in the context of HFpEF. Therapeutic strategies that target these dysregulated pathways, including IL-6 inhibition, mitochondrial antioxidants, and chaperone-mediated protection, may enhance myocardial function in HFpEF patients with T2DM. Addressing these molecular dysfunctions could facilitate the development of novel interventions specifically tailored to the diabetic HFpEF population.

**Graphical abstract:**

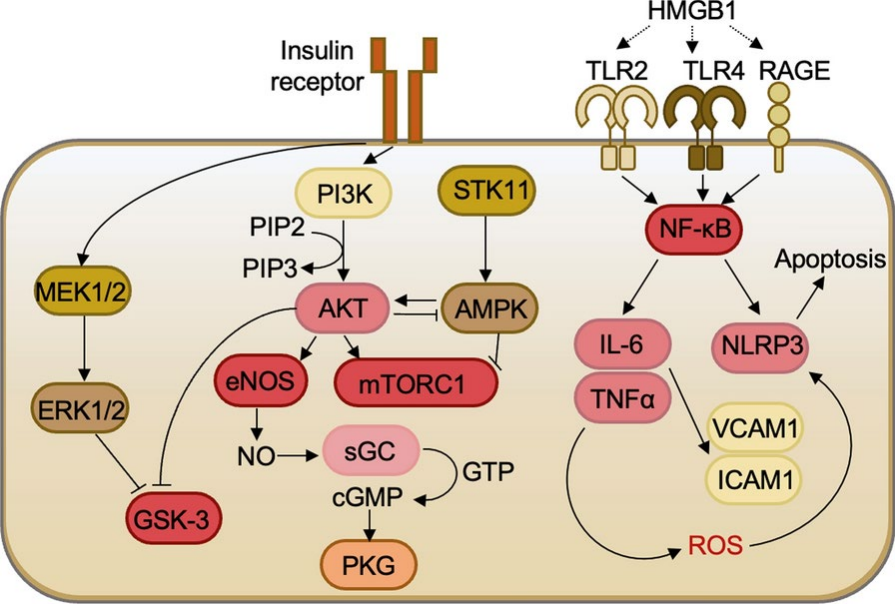

## Introduction

Type 2 diabetes mellitus (T2DM), a significant comorbidity associated with cardiovascular disease (CVD), has seen an exponential increase over the past few decades, presently impacting approximately 8% of the global population [1, 2]. Projections for the year 2019 estimated that there were 463 million cases of diabetes, with prevalence expected to double by the year 2045 [3].

Currently, CVD remains a predominant cause of mortality in individuals with T2DM, largely attributable to conditions such as coronary artery disease (CAD), ischemic cardiomyopathy, and heart failure (HF) [4–6]. Patients diagnosed with diabetes are three times more likely to develop HF, contributing to what has become a global health crisis characterized by an increasing prevalence [7]. While HF has traditionally been associated with systolic dysfunction and reduced ejection fraction—termed heart failure with reduced ejection fraction (HFrEF) [8, 9], over 50% of patients, particularly women and the elderly, exhibit symptoms such as dyspnea, fatigue, fluid retention, and exercise intolerance. This condition, referred to as heart failure with preserved ejection fraction (HFpEF), currently represents the most prevalent cause of hospitalization among individuals aged 65 years and older and is anticipated to become the most common form of heart failure [10, 11].

Evidence suggests the existence of two distinct HF phenotypes in diabetic patients: HFrEF, which is more commonly associated with Type 1 diabetes, and HFpEF, which is linked to T2DM and often accompanied by obesity, indicating a more complex underlying pathophysiology [12].

The biochemical mechanisms underlying T2DM and its association with insulin resistance result in a hyperglycemic and hypertriglyceridemic state. The impairment of glucose uptake by cardiomyocytes leads to the excessive utilization of fatty acids (FAs) as a source of energy to sustain metabolic functions. This phenomenon results in the overproduction of reactive oxygen species (ROS), reduced nitric oxide (NO) bioavailability, increased levels of pro-inflammatory cytokines, mitochondrial dysfunction, and dysregulation of autophagy and protein quality control (PQC) [13, 14].

PQC strictly regulates complex processes such as protein folding, post-translational modifications, protein trafficking, calcium (Ca^2^⁺) homeostasis, and degradation of misfolded proteins. Through these processes, newly synthesized polypeptides are transformed into functional proteins, thereby maintaining overall protein quality [15]. Disruption of protein folding, whether due to intracellular or extracellular stressors, triggers PQC activation via the unfolded protein response in the endoplasmic reticulum (UPRER), heat shock proteins (HSPs), autophagy-lysosomal pathways, and the ubiquitin–proteasome system (UPS), all of which play critical roles in maintaining protein quality [16].

Under normal physiological conditions, the expression of HSPs is relatively low. However, in the context of T2DM, pathological mechanisms lead to a significant increase in ROS levels, resulting in an excessive accumulation of oxidatively damaged proteins within cells. Under these circumstances, HSPs play a critical role in modulating oxidative stress, exhibiting various functions at multiple levels [17, 18]. As a highly conserved protein family, HSPs are essential components of the cellular stress response, serving as the primary checkpoint for PQC. Their primary functions include the elimination of misfolded proteins, the facilitation of proper protein folding, and the prevention of protein aggregation. Specifically, heat shock protein 70 (HSP70) and its co-chaperones are instrumental in targeting aberrant proteins for degradation through the proteasome and lysosome pathways. In certain instances, these chaperones possess the unique capacity to refold misfolded proteins, restoring them to their functional form [19]. Furthermore, other chaperones, such as heat shock protein 27 (HSP27) and alpha B-crystallin (αB-crystallin), are known to inhibit the apoptotic cascade, thereby promoting cellular survival and preventing apoptosis [20].

Autophagy, recognized as the second checkpoint in PQC, initiates the lysosomal digestion process, facilitating the generation of nutrients and energy necessary for sustaining cellular functions under metabolic stress. During this process, damaged macromolecules and organelles are hydrolyzed to preserve energy [21]. Autophagy with dual function has beneficial or detrimental effects on a diabetic heart [22]. Dysregulated autophagy within diabetic hearts has been associated with glucose intolerance and insulin resistance [23]. A primary focus of investigation is the role of autophagy in modulating insulin sensitivity. Evidence indicates that enhanced autophagy improves insulin sensitivity in cardiomyocytes and reduces apoptosis [24]. Increased autophagy in the heart is correlated with the production of ROS, which contributes to insulin resistance but may also function as an endogenous self-protective mechanism downstream of the PI3K/AKT insulin signaling pathway [25]. Under conditions of nutrient deficiency and low intracellular AMP/ATP ratio, AMP-activated protein kinase (AMPK) positively regulates the activation of autophagy [26]. The activation of AMPK leads to the suppression of the mammalian target of rapamycin (mTOR) activity [27]. The inhibition of mTOR activity facilitates the upregulation of stress-responsive mechanisms, including a reduction in protein synthesis, enhanced autophagy, cell cycle arrest, and DNA repair, ultimately limiting cell death [28]. The AMPK-mTOR pathway plays a crucial role in protecting against cardiovascular complications associated with diabetes through the maintenance of mitochondrial function and the suppression of oxidative stress, inflammation, excessive autophagy, and myocardial cell death or apoptosis [29–31]. Persistent hyperglycemia markedly increases the activity of the nuclear factor kappa-light-chain-enhancer of activated B-cells (NF-κB)[32]. This activation results in the heightened transcription of inflammatory genes, including pro-inflammatory cytokines such as interleukin-6 (IL-6), IL-1β, IL-18, and tumor necrosis factor (TNF-α). This chronic inflammatory state compromises the ubiquitin–proteasome system (UPS) and autophagy, ultimately contributing to cardiomyocyte damage [33]. Therefore, our study aimed to elucidate the underlying mechanisms of diabetic mellitus-induced inflammation, oxidative stress, autophagy, stress signaling and PQC system, and their contribution to the cardiac dysfunction in human HFpEF.

## Methods

### Human studies

HFpEF patients were referred for cardiac catheterization and endomyocardial biopsy procurement because of clinical suspicion of a cardiomyopathy (n = 25) see Table 1. They all had been admitted to the hospital because of worsening HF. Subsequent histological examination ruled out a cardiomyopathy/infiltrative myocardial disease. Coronary angiography showed the absence of significant coronary artery stenosis or graft vasculopathy. All patients satisfied the criteria as proposed by the European study group on HFpEF, i.e. signs and symptoms of congestive HF, LV ejection fraction (EF) ≥ 50% and abnormal LV end-diastolic pressure at rest > 12 mmHg. All patients had one or more predisposing risk factors for diastolic LV dysfunction. Endomyocardial biopsies were obtained from the LV and were snap frozen in liquid nitrogen and stored at -80 °C until processing. Samples were collected from HFpEF diabetic patients (n = 10, average age 70 years) and non-diabetic patients (n = 15, average age 75 years). In total, we have used 25 biopsies throughout the manuscript in different experiments. Due to the limited availability of the tissues, we did not use the same biopsies in the all experiments. Clinical, echocardiographic, and medication characteristics are provided in Table 1. Tissues were collected in a cardioplegic solution and stored in liquid nitrogen until use. All samples were obtained with informed consent and approval from the local Ethics Committee. All procedures were performed according to the Declaration of Helsinki and were approved by the local ethics committee. Biopsies were obtained for the primary purpose of diagnosis following Ethics Committee approval numbers WO18.026, 20–6976 BR, 20–6976-1- BR and informed consent.

**Table 1:** Clinical, echocardiographic, and medication characteristics of HFpEF patients with and without diabetes mellitus

HF phenotype	HFpEF-non DM	HFpEF-DM
number of the Patient	n = 15	n = 10
Age	75.27 ± 2.06	70.88 ± 2.08
Gender % (M = male)	M = 13.3%	M = 10%
BMI (kg/m^2^)	32.96 ± 2.26	32.35 ± 2.16
SBP (mmHg)	145.73 ± 4.47	146.38 ± 7.16
DBP (mmHg)	77.13 ± 2.24	75.75 ± 2.96
Hb (g/dL)	8.62 ± 0.19	7.81 ± 0.25
Creatinin (mmol/L)	80.73 ± 5.35	115.38 ± 29.54
eGFR (CKD-EPI) (ml/min/1.73m^2^)	64.86 ± 3	54.29 ± 11.54
NTproBNP (pmol/l)	34.07 ± 6.47	53.88 ± 24.88
CRP (mg/L)	4.26 ± 1.11	2.83 ± 0.93
LVMI (g/m^2^)	84.86 ± 5.79	91.63 ± 8.21
LVEF (%)	59.69 ± 2.86	59.64 ± 2.94
E/e' mean ratio	11.95 ± 0.89	14.04 ± 1.3
LAVI max (ml/m^2^)	53.13 ± 5.75	45.8 ± 3.59
Loop diuretic	9/15	5/10
Thiazide diuretic	2/15	0/10
MRA	6/15	4/10
Bblocker	6/15	6/10
ACEi	2/15	1/10
ARB	8/15	4/10
SGLT2i	14/15	7/10
Statin	8/15	7/10
Oral anti-glycaemic agent	0/15	6/10
Insulin	0/15	4/10

Number of patient (n), Age (years), gender % (M = male), Body mass index (BMI, kg/m^2^), Systolic blood pressure (SBP, mmHg), Diastolic blood pressure (DBP, mmHg), Hemoglobin (Hb, g/dL), Creatinine (µmol/L), Estimated glomerular filtration rate (eGFR, mL/min/1.73 m^2^), N-terminal pro-brain natriuretic peptide (NT-proBNP, pmol/L), C-reactive protein (CRP, mg/L), Left ventricular mass index (LVMI, g/m^2^), Left ventricular ejection fraction (LVEF, %), E/e' mean (ratio of early mitral inflow velocity to mitral annular early diastolic velocity), Maximum left atrial volume index (LAVI max, mL/m^2^), Loop diuretic, Thiazide diuretic), Mineralocorticoid receptor antagonist (MRA), Beta-blocker (Bblocker), Angiotensin-converting enzyme inhibitor (ACEi), Angiotensin II receptor blocker (ARB), Sodium-glucose cotransporter-2 inhibitor (SGLT2i), Statin, Oral anti-glycaemic agent (OAG), Insulin. Values are given as mean ± SEM or number

### Force measurements on isolated de-membranated cardiomyocytes:

Force measurements were conducted on single-skinned cardiomyocytes (n = 30–36/5 cardiomyocytes/hearts per group) as described before [34, 35]. To summarize, LV samples were de-frozen in a relaxing solution (containing in mM: 1.0 free Mg^2+^; 100 KCl; 2.0 EGTA; 4.0 Mg-ATP; 10 imidazole; pH 7.0), mechanically disrupted, and incubated for 5 min in a relaxing solution supplemented with 0.5% Triton X-100 (all from Sigma-Aldrich). The cell suspension was washed 5 times in relaxing solution. Single cardiomyocytes were selected under an inverted microscope (Zeiss Axiovert 135, 40 × objective; Carl Zeiss AG Corp, Oberkochen, Germany) and attached with silicone adhesive between a force transducer and a high-speed length controller (piezoelectric motor) as part of a "Permeabilized Myocyte Test System" (1600A; with force transducer 403A; Aurora Scientific, Aurora, Ontario, Canada).

Cardiomyocyte Ca^2+^-independent passive force (F_passive_) was measured in relaxing buffer at room temperature (RT) within a sarcomere length (SL) range between 1.8 and 2.4 μm. Force values were normalized to myocyte cross-sectional area calculated from the diameter of the cells, assuming a circular shape. Subsequently cardiomyocytes were incubated for 30 to 40 min in relaxing solution supplemented with either (1) an inhibitor of interleukin 6 (IL-6) (Siltuximab, 0.015 mL/L, EUSA Pharma Ltd, Hemel Hempstead, Hertfordshire, UK), (2) the mitochondria-targeted superoxide dismutase mimetic MitoTEMPO (10 µM; Sigma-Aldrich Merck KGaA, St. Louis, MO, USA), (3) heat shock protein (Hsp) (1 mg/mL; Sigma-Aldrich Merck KGaA, St. Louis, MO, USA), or (4) HSP70 (1 mg/mL; Sigma-Aldrich Merck KGaA, St. Louis, MO, USA).Cardiomyocyte F_passive_ was thereafter measured within a SL range between 1.8 and 2.4 μm as described above. All treatments with MitoTEMPO, HSPs, and IL-6 inhibitor were done in intact tissues.

### Immunofluorescence imaging

As we described before, immunofluorescence imaging was performed on the frozen tissue slide [15]. The frozen LV tissue slides were air-dried for 10 min, rehydrated with PBS, and fixed with 4% paraformaldehyde in phosphate-buffered saline (PBS) for 5 min. The slides were then washed three times for 5 min with PBS. Next, the tissue was incubated with wheat germ agglutinin (WGA 555 conjugate, invitogen, W32464) for 5 min, followed by another three washes for 5 min with PBS (5 min each). Next, the slides were blocked with 5% BSA/PBS and 0,1% Triton for 1 h at room temperature. After washing three times with PBS, the primary antibody was diluted in 5% BSA/PBS and incubated overnight at 4 °C (Table 2). After incubation with the primary antibody and washing with PBS three times, the slides were incubated with the secondary antibody diluted in 5% BSA/PBS overnight at 4 °C (Table 2). The slides were then washed again three times with PBS and then covered and sealed with Mowiol mounting medium and ultrathin glass coverslips overnight at 4 °C. For the imaging, the confocal laser scanning microscopy (cLSM) (Nikon Eclipse Ti-E inverted Microscope System, Nikon Instruments, Nicon Corp, Shinagawa, Tokyo Japan) was used.

**Table 2:** Corresponding antibody for Immunofluorescence imaging

Antibody	Product No	Company	Dilution
IL6	P620	Thermo Fisher Scientific	1:150
IL6R	Ab275982	Abcam	1:150
NE	Sc-55549	Santa Cruz Biotechnology	1:100
MPO	14569s	Cell Signaling Technology	1:100
Anti-mouse	7076S	Cell Signaling Technology	1:10000
Anti-Rabbit	7074S	Cell Signaling Technology	1:10000
Alexa Fluor Plus 488 (Anti-Rabbit)	A32731	Thermo Fisher Scientific	1:150
Alexa Fluor Plus 647 (Anti-Mouse)	A32728	Thermo Fisher Scientific	1:150

### Protein analysis with western blot

SDS-gel electrophoresis was performed to detect proteins as we described before [36, 37]. The LV tissue samples were solubilized in sample buffer (8% SDS, 40% Glycerin, 200 mM DTT, 0,4% Bromphenol blue, 253 mM Tris/HCl pH 6,8). The samples were then boiled for 3 min at 97 °C, followed by centrifugation for 3 min at 13,000 rpm. Protein concentration was determined using the Pierce 660 nm Protein Assay (22660 Thermo Fisher). Samples were separated 10–15% SDS-PAGE. Gels were run at 90 V for 20 min followed by 125 V for about 90 min (running buffer; 24.76 mM Tris, 192 mM Glycine, 3.47 mM SDS). After electrophoresis, proteins were transferred onto a PVDF membrane (Immobilon-P transfer membrane, 0.45 μm, Merck Millipore) using the Trans-Blot Turbo system from Bio-Rad(Anode buffer 300 mM Tris, 100 mM Tricin, pH 8.8; cathode buffer 300 mM Aminohexanoic Acid, 30 mM Tris, pH 8.7 pH). The membrane was activated with methanol for 1 min before the transfer. Afterwards, the blots were blocked with 5% BSA in TBST (20 mM TRIS, 137 mM NaCl, 0.1% Tween 20) for 1 h at RT and subsequently with primary antibody overnight at 4 °C. After washing with TBST, the secondary antibody was applied for 1 h at room temperature in 5% BSA in TBST and the signal was detected with the ChemiDoc MP imaging system from Bio-Rad. For the analysis, the signal was normalized to GAPDH using Multi Gauge V3.2 and BioRad ImageLab V6.1 software (Table 3).

**Table 3:** Corresponding Antibody list for western blot experiment

Antibody	Product NO	Company	Dilution
TNF-α	AMC3012	Invitrogen	1:500
IL-6	Sc.32296	Santa Cruz Biotechnology	1:1000
IL-6 Receptor	MA5-29721	Invitrogen	1:1000
eNOS	32027	Cell Signaling Technology	1:1000
HSP-27	ab2790	Abcam	1:1000
HSP-70	ab2787	Abcam	1:1000
PKG	13511	Cell Signaling Technology	1:1000
p38 MAPK	9212S	Cell Signaling Technology	1:1000
NF-κB p65(D14E12)	8242	Cell Signaling Technology	1:1000
ERK (1/2) p44/42 MAPK	4695	Cell Signaling Technology	1:1000
AKT pan (C67E7)	4691	Cell Signaling Technology	1:1000
Phospho-AKT (S473) (736E11)	3787	Cell Signaling Technology	1:1000
AMPK (D5A2)	5831	Cell Signaling Technology	1:1000
Phospho-AMPK (Thr172) (40H9)	2535	Cell Signaling Technology	1:1000
mTOR (7C10)	2983	Cell Signaling Technology	1:1000
Phospho-mTOR (Ser2448)	2971	Cell Signaling Technology	1:1000
NLRP3 (D4D8T)	15101	Cell Signaling Technology	1:1000
PI3 Kinase p110α (C73F8)	4249	Cell Signaling Technology	1:1000
GSK-3β	MA5-1509	Thermo Fisher Scientific	1:1000
GAPDH-anti mouse	97166	Cell Signaling Technology	1:10000
GAPDH-anti rabbit	2118	Cell Signaling Technology	1:10000

### Quantification of tissue oxidative and metabolic stress and inflammation

Myocardial levels (n = 10 samples/group) of inflammatory and oxidative stress markers were measured with enzyme-linked immunosorbent assay (ELISA) and colorimetric assay kits according to the manufacturer's instructions. The following kits were used in this study: high mobility group box protein 1 (HMGB1) ELISA kit (MBS701378; MyBioSource), Toll-like receptor 2 (TLR2) ELISA kit (RAB0744-1KT; Sigma-Aldrich), TLR4 ELISA kit (RAB1088-1KT; Sigma-Aldrich), receptor for advanced glycation end products (RAGE) ELISA Kit (ab190807; Abcam), NOD-like receptor protein 3 (NLRP3) ELISA Kit (ab190807; Abcam), IL-6 ELISA kit (ab100772; Abcam) and 3-nitrotyrosine ELISA kit (ab116691; Abcam), lipid peroxidation (malondialdehyde; LPO) ELISA Kit (ab118970; Abcam), tumor necrosis factor (TNFa) ELISA kit (ab108913; abcam), Intercellular adhesion molecule 1 (ICAM-1) ELISA kit(ERICAM1; Thermo Fisher), vascular cell adhesion molecule 1 (VCAM-1) ELISA kit (KHT0601; Thermo Fisher.

Stable hydrogen peroxide (H2O2) accumulation was also assessed in myocardial homogenates and mitochondrial fractions using a colorimetric assay (Sigma Aldrich). The GSSG/GSH ratios, LPO and H2O2 levels were determined both in myocardial homogenates and in the mitochondrial fraction. For fractionation, a subcellular protein fractionation kit (78840; Thermo Fisher Scientific) was used according to the manufacturer's instructions.

### Quantification of nitric oxide (NO) level 

The levels of NO were measured by means of a colorimetric assay kit (BioVision Inc, Milpitas, CA, USA) providing the measurement of total nitrate/nitrite as previously described [36, 38]. NO production was measured in tissue homogenates. Briefly, LV tissue samples (n = 10 LV samples/group) were treated with trichloroacetic acid (8 g in 80 mL acetone; Sigma-Aldrich) and washed with 1 ml 0.2% dithiothreitol (DTT). Tissue samples were homogenized in 1% SDS sample buffer (Tri-distilled water: 8.47 mL; glycerol: 2.1 mL; 10% SDS: 1.4 mL; 0.5 M Tris–HCl (pH 6.8): 1.75 ml; brome-phenol blue: 0.28 mL; DTT: 32.4 mg; all from Sigma-Aldrich). After homogenization, tissue samples underwent sonication and were subsequently centrifuged at 14,000 g for 15 min at 2–8 °C. Supernatants containing equal amounts of total protein were analysed for NO concentration. sGC was measured in 100 mg tissues, which were rinsed and homogenised in PBS and stored overnight in − 20 °C.

### Measurement of soluble guanylyl cyclase (sGC) activity 

The activity of sGC of was assessed by means of a colorimetric assay kit (MyBioSource) as we described before [36]. In brief, 100 mg of tissue (n = 10 LV samples/group) were rinsed and homogenised in PBS and stored overnight in − 20 °C. These tissue samples underwent sonication and were subsequently centrifuged at 14,000 g for 15 min at 2–8 °C. Supernatants containing equal amounts of total protein were analysed for sGC activity according to manufacturer's instructions. The absorbance of samples was measured at 570 nm using a plate reader.

### Measurement of myocardial cyclic guanosine monophosphate (cGMP) level

Myocardial cGMP levels were measured according to previous protocols [39, 40]. Briefly, cGMP was determined in LV homogenates (n = 10 samples/group) by means of a parameter cGMP assay immunoassay kit (R&D Systems, Minneapolis, MN, United States), in which cGMP present in the homogenate competes with a fixed amount of HRP-labeled cGMP for sites on a rabbit polyclonal antibody. These homogenates were diluted in cell lysis buffer, and 100 µL of 0.025 µg/µL protein aliquots were measured according to the manufacturer's instructions. The results of duplicate determinations were averaged and expressed as μg/μl.

### Measurement of myocardial protein kinase G (PKG) activity 

LV tissue samples (n = 10 samples/group) were homogenized in 25 mM Tris–HCl (pH 7.4), 1 mM EDTA, 2 mM EGTA, 5 mM DTT, 0.05% Triton X-100, and protease inhibitor cocktail (all from Sigma-Aldrich) and centrifuged for 5 min. Supernatants containing equal amounts of total protein were analysed for PKG activity as described previously [39]. In brief, reaction mixtures were incubated at 30 °C for 10 min. Reaction mixtures contained 40 mM Tris–HCl (pH 7.4), 20 mM Mg(CH3COO)2, 0.2 mM [32P] adenosine triphosphate (ATP) (500–1,000 cpm pM–1; Amersham PLC, Little Chalfont, UK), 113 mg/mL heptapeptide (RKRSRAE) and 3 μM cGMP (both from Promega Corp, Madison, WI, USA), and a highly specific inhibitor of cyclic adenosine monophosphate-dependent protein kinase (5–24; Calbiochem, San Diego, CA, USA). The reaction was terminated by spotting 70 μL onto Whatman P-81 filters (MACHEREY–NAGEL). Samples were subsequently incubated and washed with 75 mM H3PO4 for 5 min to remove unbound ATP. Filters were then washed with 100% ethanol and air dried before quantification. PKG activity was quantified using a Wallac 1409 Liquid Scintillation Counter (Hidex Oy, Turku, Finland). The specific activity of PKG was expressed as pM of 32P incorporated into the substrate (pM/min/mg protein).

### RNA isolation and quantitative reverse transcription-polymerase chain reaction (qRT-PCR)

In the initial stage, human tissue samples were carefully weighed, ranging between 50 to 100 mg. The entire procedure was executed to preserve RNA activity under the application of liquid nitrogen. For homogenization, one stainless steel bead and 1 mL of QIAzol Lysis Reagent (Qiagen, 79306) were added to each tissue sample on ice under a safety bench. The samples were homogenized in the Precellys Evolution Touch Homogenizer (Berlin Technologies) for 2 cycles of 5 min at 5000 rpm with cooling. After incubation on ice for 5 min, the samples were centrifuged at 13,000 rpm for 10 min at 4 °C. The supernatant was carefully transferred to a clean tube, and 200 μL of chloroform was added. The mixture was vortexed, incubated at RT for 3 min, and then centrifuged at 13,000 rpm for 15 min to facilitate phase separation. The RNA-containing aqueous phase was subsequently carefully transferred to a new tube.

Next, 360 μL of 100% isopropanol and 1 μL of Glycogen azure (Sigma Aldrich, G5510) were added, mixed, and incubated for 10 min at RT. After centrifugation at 13,000 rpm for 10 min, the supernatant was removed, and the RNA pellet was washed four times with 1 mL of 75% ethanol, followed by centrifugation at 7500 rpm for 5 min each time. After the final wash, the pellet was air-dried and resuspended in 10 μL of RNase-free water. RNA concentrations were measured using spectrophotometry (Implen Nanophotometer® N60).

### cDNA synthesis

The working solution was prepared using the Sensifast cDNA synthesis kit (Bioline, BLI 65053), by mixing 4 μL of 5× TransAmp Buffer and 1 μL of reverse transcriptase/reaction. The 0.2 ml RNAse free Eppendorf tubes were filled with 5 μL of the working solution, 0.5 μg RNA per sample, and nuclease-free water to a final volume of 20 μL. Tubes were processed in a thermal cycler for cDNA synthesis. Afterward, cDNA purity was checked using spectrometry, and samples were stored at − 80 °C for subsequent qRT-PCR analysis.

The primers for the experiments were designed using the Primer-BLAST tool provided by the National Center for Biotechnology Information (NCBI). Gradient PCR measurements were carried out on the primers to determine their annulation temperature. Quality control of the primer products was performed using agarose gel electrophoresis after the PCR reactions.

Initially, the primers were dissolved, and their concentration adjusted to a final concentration of 100 μM with nuclease-free water. Reaction mixtures were prepared by combining 7.5 μL/reaction of SensiFAST SYBR® NO-ROX Kit (Bioline, BIO-98020), 4.85/reaction μL of nuclease-free water, and 0.075 μL/reaction of forward and reverse primer solutions, respectively. Subsequently, 12.5 μL of the reaction mixture was dispensed into each well of a 384-well LC Multiwell Plate (Roche), followed by 2.5 μL of the respective samples in duplicates. The plate was sealed, centrifuged at 1500 rpm for 2 min, and placed onto the LightCycler® 480 qPCR machine (Roche). The program was initiated using the LightCycler® 480 SW 1.5.1 software, with data analysis completed after approximately 1.5 h. The results were calculated using the 2 − ΔΔCp method. Peptidylprolyl isomerase A (PPIA) and ribosomal protein L13A (RPL13A) functioned as housekeeping genes (Table 4).

**Table 4:** Corresponding forward and revers primers list used for qRT-PCR

Target gene	Accession Number	Primer Name	Sequence	Manufacturer
MTOR	NM_004958.4	hsa_MTOR_fw	GAAGCCGCGCGAACCT	Bio-Science Kft
		hsa_MTOR_rev	ATTCCGGCTCTTTAGGCCAC	
MAPK1	NM_002745.5	hsa_MAPK1_fw	ATTACGACCCGAGTGACGAG	Bio-Science Kft
		hsa_MAPK1_rev	CTCTGAGCCCTTGTCCTA	
MAPK3	NM_002746.3	hsa_MAPK3_fw	CATGCTGAACTCCAAGGGCTA	Bio-Science Kft
		hsa_MAPK3_rev	GATCCAGGTAGTGCTTGCCA	
AMPK	NM_006251.6	hsa_AMPK_fw	CGGCAAAGTGAAGGTTGGC	Bio-Science Kft
		hsa_AMPK_rev	CCTACCACATCAAGGCTCCG	
NOS3	NM_000603.5	hsa_NOS3_fw	CCGGAACAGCACAAGAGTT	Bio-Science Kft
		hsa_NOS3_rev	CCCTGCACTGTCTGTGTTAC	
PRKG1	NM_006258.4	hsa_PRKG1_fw	AAGAGCCCACAGTCCAAGG	Bio-Science Kft
		hsa_PRKG1_rev	ATCTGCGACAGCTCCAAGTT	
STK11	NM_000455.5	hsa_STK11_fw	GAGGCCAGTCACAATGGACA	Bio-Science Kft
		hsa_STK11_rev	CCTGGACACGGGCTGC	

### Statistics

We used estimation plots which show on the left axis the data as box and whisker plots (median, 25th to 75th percentiles, minimum and maximum) in order to present individual points in each group. The effect size meaning the difference between the means (mean ± SEM) is shown on the right axis. The *P*-values are from unpaired Student´s t-test; * P < 0.05, ** *P* < 0.01, *** *P* < 0.001. For the analysis of force measurements, which involves parametric data comparing more than two groups, one-way ANOVA was used. *P*-values were adjusted for multiple comparisons using the Tukey method; significant comparisons include * P < 0.05 HFpEF-DM vs. HFpEF + DM, ‡ *P* < 0.05 HFpEF-DM vs. HFpEF-DM after treatment, † *P* < 0.05 HFpEF + DM vs. HFpEF + DM after treatment by one-way ANOVA. For analysis of proportions, Fisher's exact test was used. The analysis was performed using GraphPad Prism 10. *P*-values are two-sided and considered statistically significant if *P* < 0.05.

## Results

### Increased inflammatory signaling pathways and inflammasome activation in HFpEF patients with diabetes mellitus

**Fig. 1:**
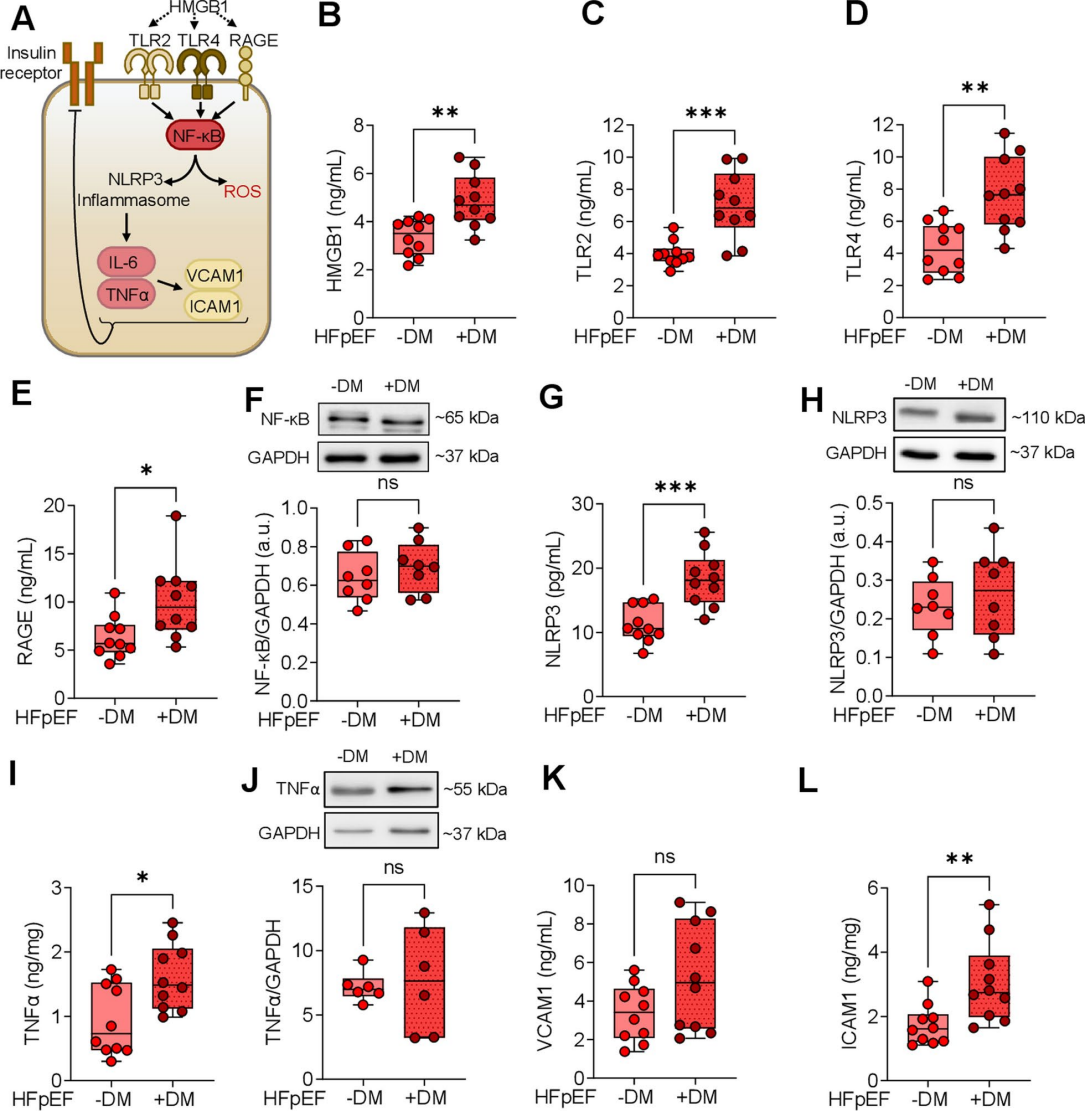
Signaling Pathways of Inflammation in Left Ventricular Biopsies from Patients with HFpEF, with (+ DM) and without Concomitant Diabetes (-DM). **A** Schematic representation of intracellular inflammatory signaling pathways. HMGB1: high mobility group box protein 1; ICAM1: intercellular adhesion molecule; IL-6: interleukin 6; NF-κB: nuclear factor kappa B; NLRP3: NOS-like receptor protein 3; RAGE: receptor for advanced glycation end products; ROS: reactive oxygen species; TLR: Toll-like receptor; TNFα: tumour necrosis factor α; VCAM1: vascular cell adhesion protein. **B** HMGB1 levels. **C**. TLR2 levels. **D** TLR4 levels. **E** RAGE levels. **F.** NF-κB expression over GAPDH. **G** NLRP3 levels. **H** NLRP3 expression over GPADH. **I** TNFα levels. **J** TNFα expression over GAPDH. **K** VCAM1 levels. **L** ICAM1 levels. Data are represented as box and whisker plots (median, 25th to 75th percentiles, minimum, and maximum (n = 6–10 samples/group). *P*-values are derived from an unpaired t-test; * *P* < 0.05, ** *P* < 0.01, *** *P* < 0.001

Inflammation accelerates the deterioration of cardiac structure and function in patients with DM, ultimately leading to heart failure. Figure 1A illustrates the inflammatory signaling pathway that serves a crucial role in linking chronic systemic inflammation and insulin resistance in patients with HFpEF, both diabetic and non-diabetic. The activation of the inflammasome is initiated by the binding of DAMPs to TLRs and the RAGE, which triggers the NF-κB signaling pathway and subsequently activates NLRP3 inflammasome [41]. Several stimuli, including oxidative stress, can promote the activation of the NLRP3 inflammasome. NFκB activation further induces the release of pro-inflammatory cytokines such as IL-6, TNFα, ICAM-1, and VCAM-1 [42, 43], which ultimately exacerbates insulin resistance in diabetic patients. In myocardial biopsies from HFpEF patients with DM, a significant upregulation of DAMPs such as HMGB1 (Fig. 1B), toll-like receptors including TLR2 (Fig. 1C) and TLR4 (Fig. 1D), as well as RAGE (Fig. 1E), was observed when compared to HFpEF patients without DM. The increased ligand-receptor binding was accompanied by significantly elevated levels of the NLRP3 inflammasome in HFpEF patients with DM compared to their non-diabetic counterparts (Fig. 1G), although no significant differences were noted in NLRP3 and NFκB protein expression levels between the two groups (Fig. 1F, H). Pro-inflammatory cytokines such as TNFα exhibited significantly increased concentration levels in the HFpEF DM group, while TNFα protein expression levels were non-significantly elevated in this group compared to the HFpEF non-DM group (Fig. 1I, J). Additionally, the concentration of the pro-inflammatory cytokine ICAM-1 was significantly increased in HFpEF DM patients compared to HFpEF non-DM patients, whereas VCAM-1 levels were non-significantly elevated in HFpEF DM patients (Fig. 1L, K), indicating a pronounced presence of inflammation in HFpEF patients with DM.

### Elevated IL6 levels and improvement in cardiomyocyte passive stiffness upon IL6 inhibitor treatment

**Fig. 2:**
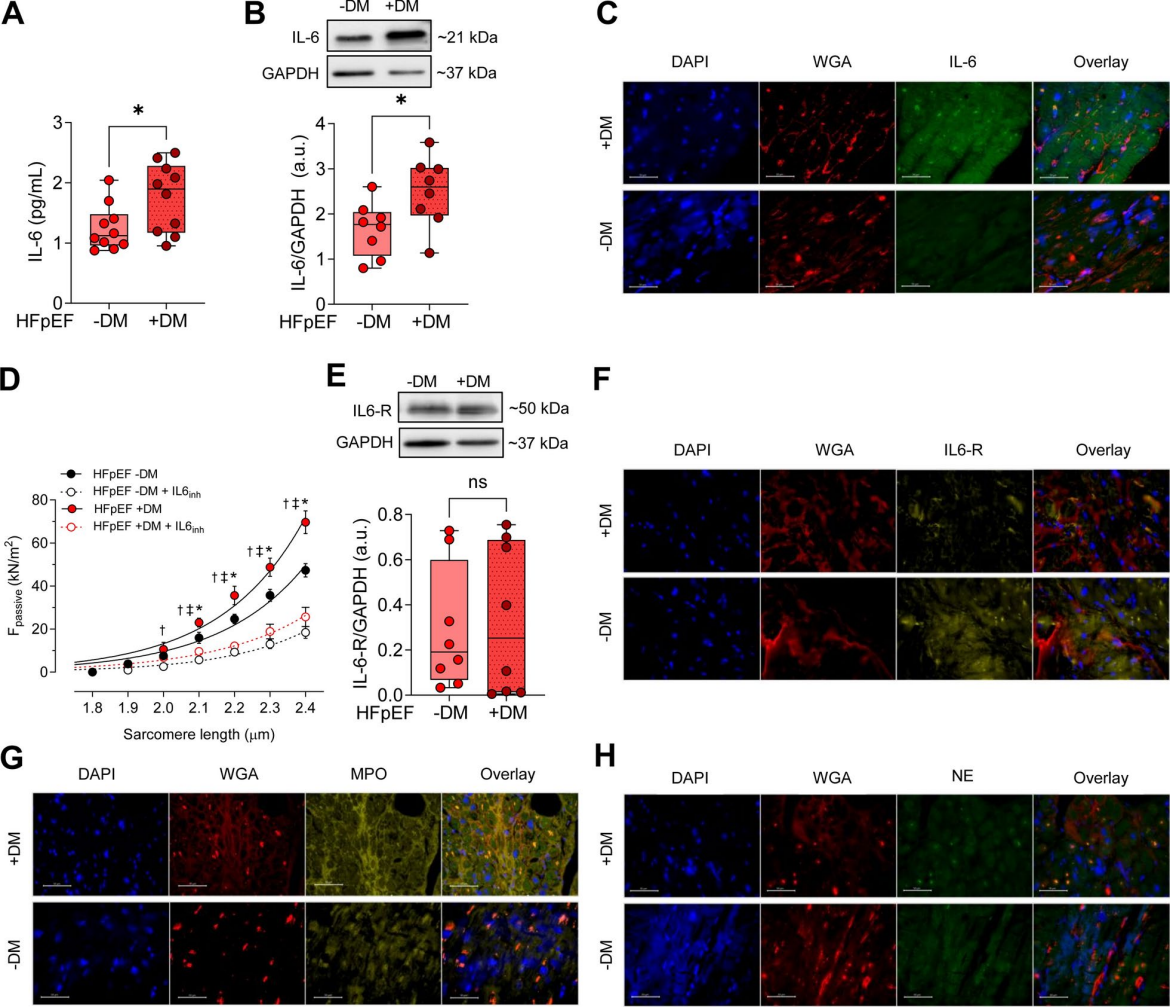
Inflammatory Markers in Left Ventricular Biopsies from Patients with HFpEF, with (+ DM) and without Concomitant Diabetes (−DM). **A** Interleukin-6 (IL-6) levels. **B** IL-6 expression over GAPDH. **C** Immunoflourescence staining of IL-6. DAPI staining (blue) and WGA (anti-wheat agglutinine 555 conjugate, red) staining are used for nucleic acids and membranes. **D** F_passive_ at sarcomere length 1.8–2.4 µm in the presence or absence of IL-6 inhibitor. Curves are second-order polynomial fits to the means (± SEM; *n* = 30–36/5 cardiomyocytes/heart per group), * *P* < 0.05 HFpEF -DM vs. HFpEF + DM, ^‡^*P* < 0.05 HFpEF -DM vs. HFpEF -DM after IL-6_inh_, † *P* < 0.05 HFpEF + DM vs. HFpEF + DM after IL-6_inh_ treatment by one-way ANOVA. *P*-values were corrected for multiple comparisons by the Tukey method. **E** IL-6 receptor (IL6-R) expression over GAPDH. **F** Immunoflourescence staining of IL6-R. **G** Immunoflourescence staining of myeloperoxidase (MPO). **H** Immunoflourescence staining of neutrophil elastase (NE). Panels **A + B + E**. Data are represented as box and whisker plots (median, 25th to 75th percentiles, minimum, and maximum (n = 6–10 samples/group). *P*-values are derived from an unpaired t-test

The concentration and protein expression levels of IL6 were significantly upregulated in HFpEF DM patients compared to HFpEF non-DM patients (Fig. 2A, B) and clearly in line with enhanced fluorescence intensity shown by immunohistochemistry staining in HFpEF DM patients compared to HFpEF non-DM patients (Fig. 2C).

Emerging evidence suggests that chronic systemic inflammation in patients with HFpEF significantly impacts various cardiac structural and functional alterations. These alterations encompass concentric remodeling, increased cardiomyocyte stiffness (F_passive_), myocardial hypertrophy, and interstitial fibrosis [44]. Consistent with these findings, we observed a significant increase in F_passive_ in isolated skinned cardiomyocytes obtained from HFpEF DM patients compared to HFpEF non-DM patients, particularly at optimal resting sarcomere lengths (SL) ranging from 2.0 to 2.4 µm (Fig. 2D). To investigate whether the observed increase in inflammatory markers correlates with these findings, we administered an IL6 inhibitor to the isolated skinned cardiomyocytes derived from both HFpEF DM and HFpEF non-DM patients. Notably, treatment with the IL6 inhibitor significantly reduced F_passive_ in HFpEF DM cardiomyocytes at SLs of 2.1 to 2.4 µm. Additionally, administration of the IL6 inhibitor resulted in a decrease in F_passive_ among HFpEF non-DM cardiomyocytes, demonstrating levels lower than those observed in HFpEF DM following IL6 inhibitor treatment. IL6 receptor expression, as determined by western blot analysis and immunohistochemistry staining, was slightly elevated in the HFpEF DM group; however, this difference did not reach statistical significance (Fig. 2E, F). Furthermore, MPO and NE showed higher fluorescence intensity by immunohistochemistry staining in HFpEF DM patients (Fig. 2G, H).

### Diminished NO-sGC-cGMP-PKG pathway in HFpEF DM patients

**Fig. 3:**
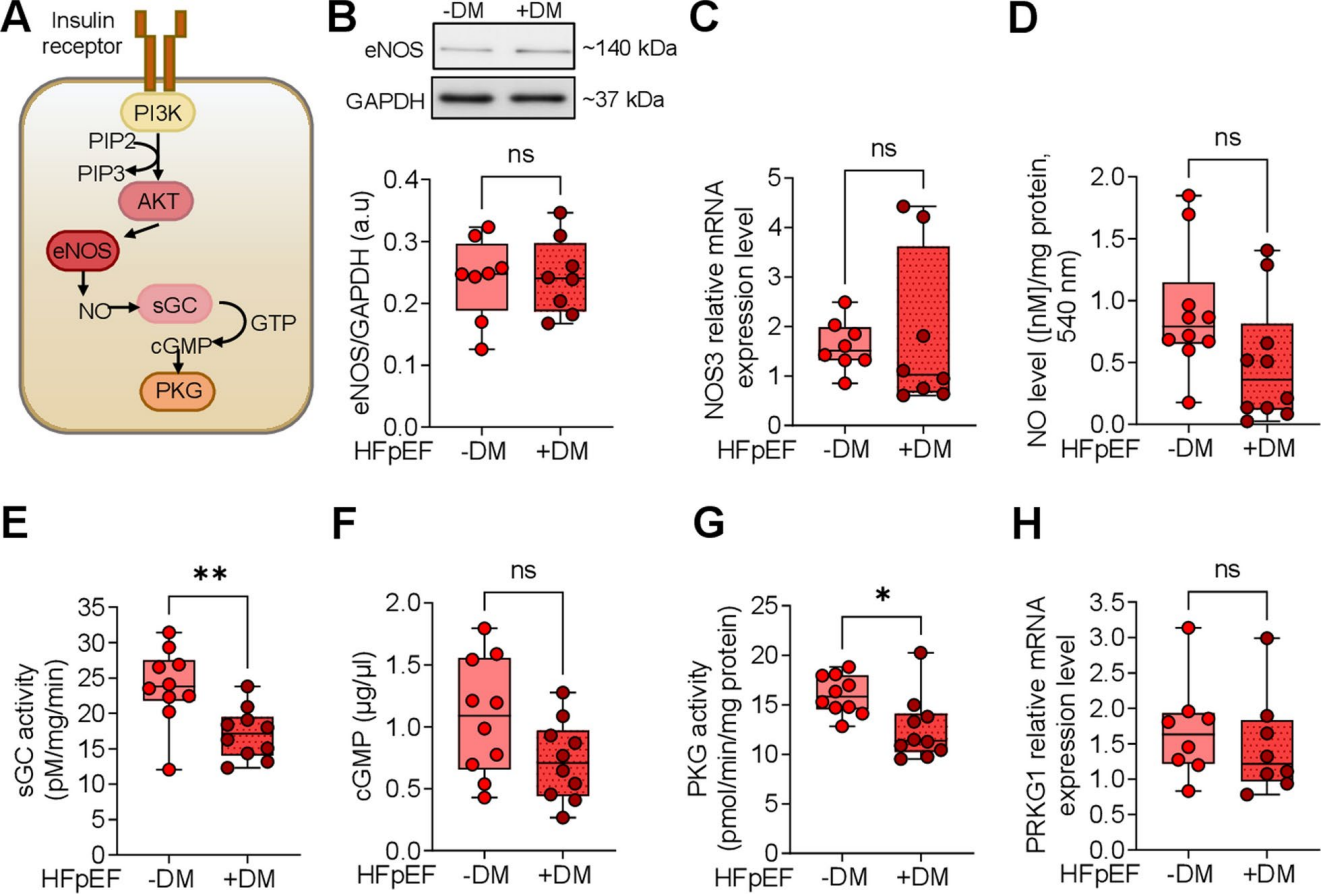
Protein kinase G Signaling Pathway in Inflammation in Left Ventricular Biopsies from Patients with HFpEF, with (+ DM) and without Concomitant Diabetes (-DM). **A** Schematic representation of the PKG signaling pathway. AKT: AKT serine/threonine kinase; cGMP: cyclic guanosine monophosphate; eNOS, endothelial nitric oxide synthase; GTP: guanosine triphosphate; NO: nitric oxide; PI3K: phosphatidylinositol-3-kinase; PIP2: phosphatidylinositol-4,5-bisphosphate; PIP3: phosphatidylinositol-3,4,5-trisphosphate; PKG: protein kinase G; sGC: soluble guanylyl cyclase. **B** Expression of eNOS over GAPDH. **C** NOS3 mRNA level. **D** NO level. **E** Activity of sGC**. F** Myocardial cGMP level. **G** PKG activity**. H** PRKG1 mRNA level. Data are represented as box and whisker plots (median, 25th to 75th percentiles, minimum, and maximum (n = 8–10 samples/group). *P*-values are derived from an unpaired t-test; * *P* < 0.05, ** *P* < 0.01

Figure 3A illustrates the eNOS-NO-sGC-cGMP-PKG pathway and its contribution to oxidative stress and insulin resistance in HFpEF patients with and without diabetes (Fig. )3B–H). The expression level of eNOS remained unchanged in HFpEF DM and HFpEF non-DM patients (Fig. 3B). In HFpEF DM patients, NO bioavailability was reduced, though not to a statistically significant level (Fig. 3D), while NOS3 mRNA levels were increased non-significantly in the HFpEF DM group (Fig. 3C). The reduction in NO bioavailability was accompanied by a significant decrease in sGC activity, reduced cGMP levels, and subsequently a significant reduction in PKG activity observed in HFpEF DM patients compared to HFpEF non-DM patients (Fig. 3E, F, G). However, at the mRNA expression level, PKG exhibited no significant differences between HFpEF diabetic and non-diabetic groups (Fig. 3H).

### Increased oxidative stress markers and antioxidative treatment mediated reduction in passive stiffness in HFpEF DM and HFpEF non-DM patients

**Fig. 4:**
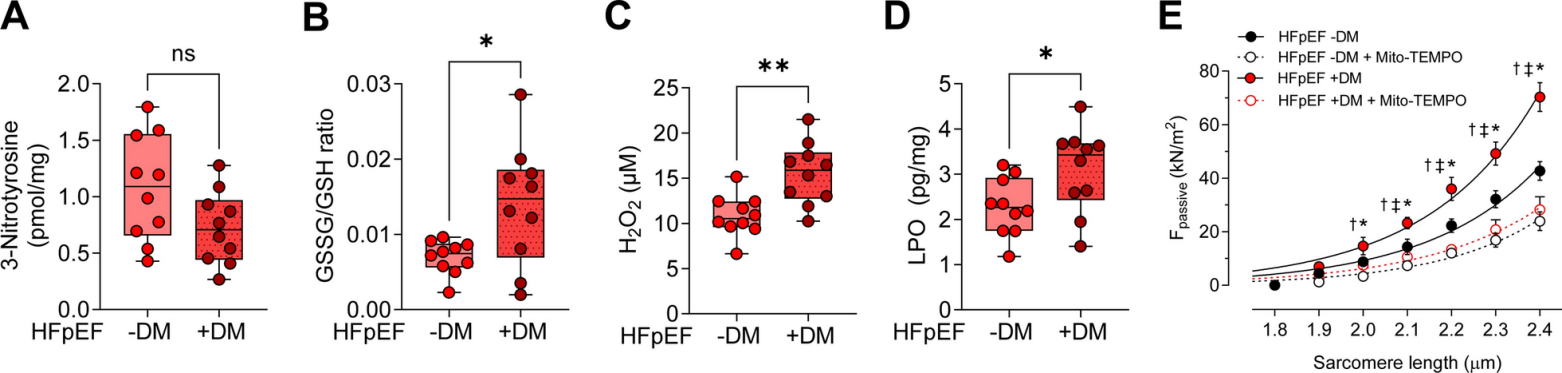
Oxidative Stress Markers in Inflammation in Left Ventricular Biopsies from Patients with HFpEF, with (+ DM) and without Concomitant Diabetes (-DM). **A** 3-Nitrotyrosine levels. **B** Ratio of myocardial oxidized glutathione (GSSG) over reduced glutathione (GSH). **C** Myocardial hydrogen peroxide (H_2_O_2_) levels. **D** Myocardial lipid oxidation (LPO) levels. Data are represented as box and whisker plots (median, 25th to 75th percentiles, minimum, and maximum (n = 10 samples/group). *P*-values are derived from an unpaired t-test; * *P* < 0.05, ** *P* < 0.01. **E** F_passive_ at sarcomere length 1.8–2.4 µm in the presence or absence of Mito-TEMPO. Curves are second-order polynomial fits to the means (± SEM; *n* = 30–36/5 cardiomyocytes/heart per group), * *P* < 0.05 HFpEF -DM vs. HFpEF + DM, ^‡^*P* < 0.05 HFpEF -DM vs. HFpEF -DM after Mito-TEMPO, † *P* < 0.05 HFpEF + DM vs. HFpEF + DM after Mito-TEMPO treatment by one-way ANOVA. *P*-values were corrected for multiple comparisons by the Tukey method

It is established that oxidative stress can impair the insulin signaling pathway and is considered one of the key drivers of insulin resistance in T2DM patients. Consequently, we investigated the presence of oxidative stress by evaluating oxidative stress markers in HFpEF patients with and without DM (Fig. 4A–D). Oxidative stress markers, including the reduced/oxidized glutathione ratio (GSSG/GSH), hydrogen peroxide (H2O2), LPO levels were significantly elevated in LV myocardial tissue of HFpEF DM patients compared to HFpEF non-DM patients, confirming high levels of oxidative stress in HFpEF patients with diabetes (Fig. 4B–D). Conversely, the level of 3-nitrotyrosine was reduced in HFpEF DM patients, though not significantly compared to HFpEF non-DM patients (Fig. 4A).

We further explored the impact of the mitochondria-targeted antioxidative agent Mito-TEMPO in regulating cardiomyocyte passive stiffness (F_passive_) in HFpEF DM and HFpEF non-DM isolated skinned cardiomyocytes at SLs of 1.8–2.4 µm. Administration of Mito-TEMPO significantly restored F_passive_ at SL 2.0 µm in both HFpEF DM and HFpEF non-DM cardiomyocytes, suggesting the antioxidative efficacy of Mito-TEMPO in normalizing cardiomyocyte passive tension.

### Dysregulated autophagy and metabolic stress regulators in HFpEF DM patients

**Fig. 5:**
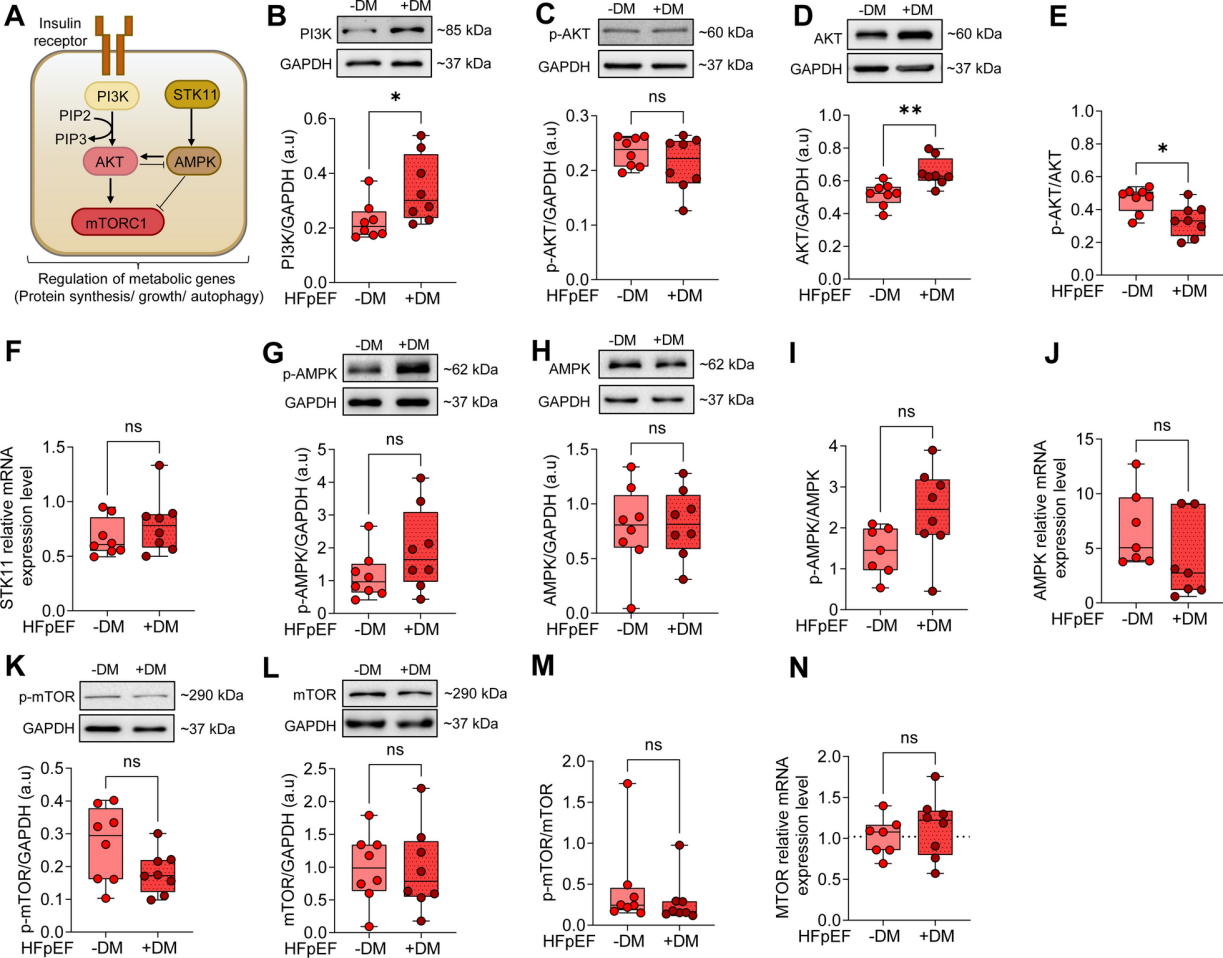
The PI3K/AKT/AMPK/mTOR Signaling Pathway Left Ventricular Biopsies from Patients with HFpEF, with (+ DM) and without Concomitant Diabetes (-DM).** A** Schematic representation of the PI3K/AKT/AMPK/mTOR signaling pathway. AMPK: AMP-activated protein kinase; AKT: AKT serine/threonine kinase; mTORC1: mammalian target of rapamycin complex 1; PI3K: phosphatidylinositol-3-kinase; PIP2: phosphatidylinositol-4,5-bisphosphate; PIP3: phosphatidylinositol-3,4,5-trisphosphate; STK11: serine/threonine kinase 11. **B** Expression of PI3K over GAPDH. **C** Phosphorylation of AKT over GAPDH. **D** Expression of AKT over GAPDH. **E** Ratio of phosphorylated over total AKT. **F** STK11 mRNA level. **G** Phosphorylation of AMPK over GAPDH. **H** Expression of AMPK over GAPDH. **I.** Ratio of phosphorylated over total AMPK. **J.** AMPK mRNA level.** K** Phosphorylation of mTOR over GAPDH. **L.** Expression of mTOR over GAPDH. **M** Ratio of phosphorylated over total mTOR. **N** mTOR mRNA level. Data are represented as box and whisker plots (median, 25th to 75th percentiles, minimum, and maximum (n = 7–10 samples/group). *P*-values are derived from an unpaired t-test; * *P* < 0.05, ** *P* < 0.01

We evaluated PI3K/Akt/mTOR and AMPK signaling pathways that play a crucial role in metabolic stress, hypertrophy, and autophagy. Figure 5A illustrates insulin-mediated key signaling pathways involved in regulating cellular metabolism, specifically focusing on protein synthesis, cellular growth, and autophagy. Cardiomyocytes abundantly exhibit insulin receptors that facilitate the binding of insulin to the insulin receptors, interact with insulin receptor substrate proteins, and activate downstream signaling pathways such as PI3K/ AKT [45]. AKT activation can phosphorylate multiple targets, including the mTOR, which plays a crucial role in coordinating cell growth, metabolism, and cell proliferation. Furthermore, AMPK is a key sensor of cellular energy status that is activated under conditions of low intracellular ATP [46]. The serine/threonine kinase STK11 is the major kinase responsible for phosphorylation and activating AMPK under stress conditions [47]. Consequently, AMPK directly phosphorylates the component regulatory protein associated with mTOR, leading to suppression of mTORC1 activity through allosteric inhibition [48].

In our study, PI3K expression demonstrated an increased trend in myocardial tissue of HFpEF DM patients compared to HFpEF non-DM patients (Fig. 5B). It was accompanied by the significantly heightened expression of AKT in HFpEF DM patients (Fig. 5D). However, no significant differences were observed in the phosphorylated status of AKT in HFpEF DM and HFpEF non-DM groups (Fig. 5C), while the activation of AKT was significantly reduced in the HFpEF DM group denoted by the p-AKT/total AKT ratio (Fig. 5E). Furthermore, the mRNA level of STK11 remained unchanged in both groups (Fig. 5F). Despite the unchanged STK11 level, the phosphorylation status of AMPK was upregulated (Fig. 5G), and the ratio of phospho/total AMPK showed an increased tendency but was non-significant in HFpEF DM patients compared to HFpEF non-DM patients (Fig. 5I), indicating the increased activation of AMPK in HFpEF diabetic hearts. Nevertheless, no alteration was observed in the AMPK protein expression level between both groups (Fig. 5H), and AMPK mRNA expression level was non-significantly reduced in the HFpEF DM group (Fig. 5J).

Additionally, we evaluated the downstream target of AKT, including the protein expression, mRNA expression, and phosphorylation levels of mTOR in HFpEF patients with and without diabetes (Fig. 5K-N). The phosphorylation status of mTOR non-significantly exhibited a downward trend in HFpEF DM patients (Fig. 5K). The protein expression level of mTOR detected by western blots was similar in both groups (Fig. 5L), while the mRNA expression level was slightly increased non-significantly in the HFpEF DM group (Fig. 5N). In alignment with the phosphorylation status of mTOR, the p-mTOR/total mTOR ratio was non-significantly reduced in HFpEF DM patients compared to HFpEF non-DM patients (Fig. 5M).

### Dysregulated MAPK signaling pathways in HFpEF DM and HFpEF non-DM hearts

**Fig. 6:**
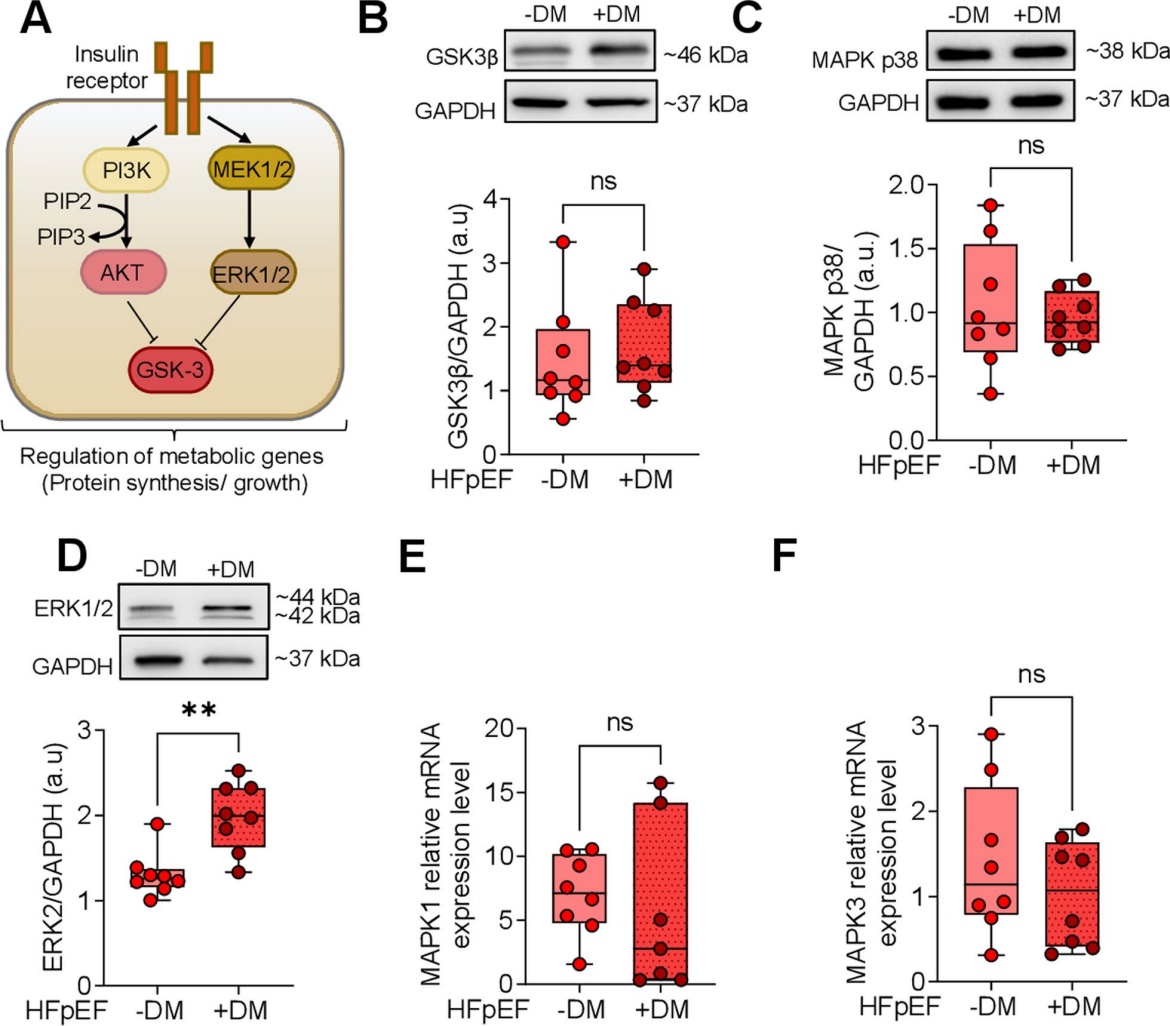
MAPKs and GSK3β in Left Ventricular Biopsies from Patients with HFpEF, with (+ DM) and without Concomitant Diabetes (-DM).** A** Schematic representation of the PI3K/AKT/ERK signaling pathway. AKT: AKT serine/threonine kinase; ERK1/2: extracellular-signal regulated kinases; GSK-3: glycogen synthase kinase 3; MEK: mitogen-activated protein kinase; PI3K: phosphatidylinositol-3-kinase; PIP2: phosphatidylinositol-4,5-bisphosphate; PIP3: phosphatidylinositol-3,4,5-trisphosphate; **B** Expression of GSK3β over GAPDH. **C** Expression of MAPK p38 over GAPDH. **D** Expression of ERK2 over GAPDH. **E** MAPK1 mRNA level. **F** MAPK3 mRNA level. Data are represented as box and whisker plots (median, 25th to 75th percentiles, minimum, and maximum (n = 6–10 samples/group). *P*-values are derived from an unpaired t-test; ** *P* < 0.01

We investigated an additional downstream pathway of insulin signaling, specifically the MAPKs pathway. Figure 6A illustrates the activation of the MAPKs pathway, which is associated with impaired insulin signaling leading to insulin resistance in the diabetic heart. The activation of the mitogen-activated protein kinase kinase 1/2 (MEK 1/2)-extracellular signal-regulated kinases 1/2 (ERK 1/2) (MEK/ERK pathway) and p38 MAPK pathways elevates the expression of GLUT1 while reducing the expression of GLUT4, resulting in increased basal glucose uptake and impaired insulin-sensitive uptake. Furthermore, the AKT activation pathway, stimulated by insulin binding, represents the primary regulatory pathway that phosphorylates glycogen synthase kinase-3 (GSK3) and inhibits its activity [49]. MAPKs can also phosphorylate and inhibit GSK3, which is associated with cardiac energy metabolism, inflammation, and fibrosis [50]. In our study, the expression level of GSK3β protein was slightly upregulated in myocardial tissue from HFpEF and DM compared to those with HFpEF and non-DM (Fig. 6B). The protein expression level of p38 MAPK was non-significantly reduced in HFpEF DM patients compared to HFpEF non-DM patients (Fig. 6C). Conversely, ERK 1/2 protein expression demonstrated a significant increase in HFpEF DM patients (Fig. 6D). The mRNA expression level of MAPK1 was markedly increased in the HFpEF DM group, while the mRNA level of MAPK3 was decreased in the HFpEF DM group compared to the HFpEF non-DM group; however, these observations did not reach statistical significance (Fig. 6E, F).

### Alterations of HSPs and HSPs mediated improvement in passive stiffness in HFpEF DM and HFpEF non-DM patients

**Fig. 7:**
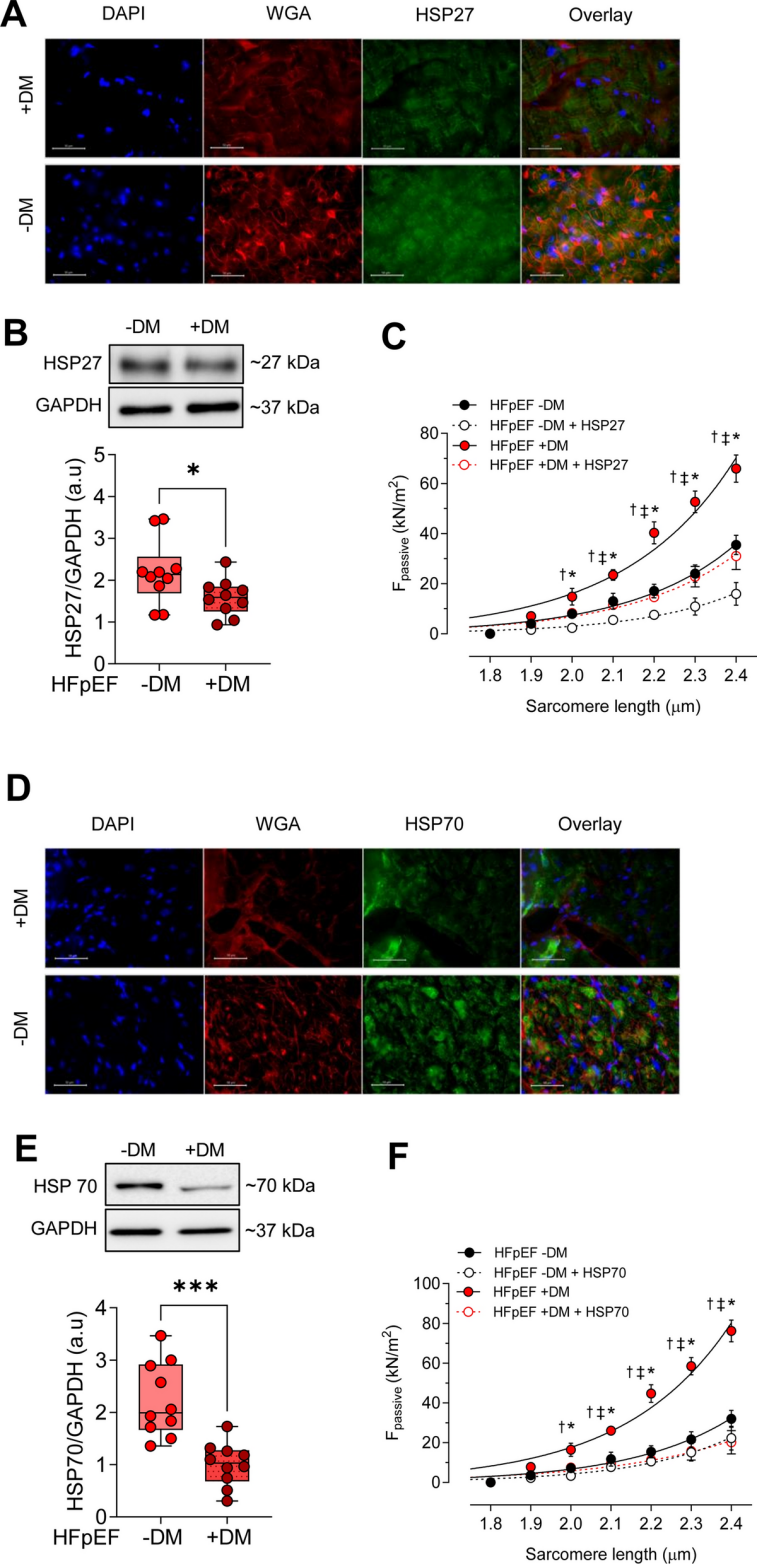
Heat-Shock Proteins (HSPs) and Cardiomyocyte Passive Stiffness in Left Ventricular Biopsies from Patients with HFpEF, with (+ DM) and without Concomitant Diabetes (-DM). **A** Immunoflourescence staining of HSP27. DAPI staining (blue) and WGA (anti-wheat agglutinine 555 conjugate, red) staining are used for nucleic acids and membranes. **B** Expression of HSP27 over GAPDH. **C** F_passive_ at sarcomere length 1.8–2.4 µm in the presence or absence of HSP27. **D** Immunoflourescence staining of HSP70. **E**. Expression of HSP70 over GAPDH. **F.** F_passive_ at sarcomere length 1.8–2.4 µm in the presence or absence of HSP70. Panels** B** + **E**: Data are represented as box and whisker plots (median, 25th to 75th percentiles, minimum, and maximum (n = 6–10 samples/group). *P*-values are derived from an unpaired t-test; * *P* < 0.05, *** *P* < 0.001. Panels **C** + **F**: Curves are second-order polynomial fits to the means (± SEM; *n* = 30–36/5 cardiomyocytes/heart per group). * *P* < 0.05 HFpEF -DM vs. HFpEF + DM, ^‡^*P* < 0.05 0.05 HFpEF -DM vs. HFpEF -DM after HSP27 or HSP70 treatment, † *P* < 0.05 HFpEF + DM vs. HFpEF + DM after HSP27 or HSP70 treatment by one-way ANOVA. *P*-values were corrected for multiple comparisons by the Tukey method

HSPs and their co-chaperons are well-defined as crucial players in PQS due to their pivotal roles in correcting misfolded proteins, directing the aberrant proteins to degradation, and inhibiting apoptosis. Therefore, we examined the alteration of heat shock proteins in HFpEF patients in both diabetic and non-diabetic groups. A significant decrease in the protein expression level of heat shock proteins, including HSP27 and HSP70, was observed in HFpEF DM samples compared to HFpEF non-DM samples (Fig. 7B, E). Reduced HSP27 and HSP70 expression is also supported by a clear reduction of the fluorescence intensity shown by immunohistochemistry staining in HFpEF DM group compared to HFpEF non-DM group (Fig. 7A, D).

To investigate whether alterations of HSPs are associated with the cardiomyocyte dysfunction, we tested the ability of HSP27, and HSP70 to reverse the elevated F_passive_ in HFpEF DM and non-DM patients. Figure 7C and F illustrate a significantly elevated F_passive_ in cardiomyocytes from HFpEF DM isolated skinned cardiomyocytes when compared to those from HFpEF patients without DM, particularly at optimal resting SL ranging from 1.8 to 2.4 µm. Administration of HSP70 corrected the increased F_passive_ at SL 2.0 µm in HFpEF DM cardiomyocyte, while no significant effects on F_passive_ was detected following HSP70 administration in HFpEF non-DM cardiomyocyte (Fig. 7F). Furthermore, a significant decrease in F_passive_ was observed in HFpEF DM cardiomyocytes following HSP27 administration, but it could not restore the F_passive_ and the reduced F_passive_ was similar to the F_passive_ of HFpEF non-DM cardiomyocyte without HSP27 treatment (Fig. 7C). In contrast, HSP27 administration restored the F_passive_ among HFpEF non-DM cardiomyocytes, exhibiting reduced levels compared to those in HFpEF DM post-treatment with HSP27(Fig. 7C).

## Discussion

This study provides evidence of increased myocardial oxidative stress and diminished PQC, as manifested by altered autophagy and HSPs. Additionally, it highlights increased apoptotic processes and dysregulated insulin signaling pathways, which are implicated in glucose uptake, metabolic stress, and post-translational modifications in HFpEF-DM compared to non-DM, all of which contribute to increased Fpassive.

### Myocardial inflammation induced by metabolic abnormalities in diabetes-heart

Diabetes-associated metabolic dysregulations trigger cytokine release and expression in cardiomyocytes [51]. Hyperglycemia, a hallmark of diabetes, was shown to upregulate HMGB1 activity in HFpEF-DM patients compared to the non-DM group. The interaction between HMGB1 and its receptors (TLR4, TLR2, and RAGE) predominantly activates NF-κB, a classic inflammatory mediator [52]. According to Mariappan et al., NF-κB activation is a critical first step in cardiac damage [53]. Romeo et al. demonstrated that in diabetic individuals, compounds that deactivate NF-κB may prove to be a potent and successful strategy for preventing cardiac apoptosis [54]. Additionally, curcumin has been shown to inhibit hyperglycemia-induced inflammation in cardiac cells in vitro and in diabetic hearts through NF-κB inactivation [55].

We observed that in the HFpEF-DM group, overexpression of NF-κB amplifies the biosynthesis and activation of multiple inflammatory cytokines (TNF-α, IL-6, ICAM, VCAM, and NLRP3 inflammasomes), which play a crucial role in pathologic inflammatory responses. Moreover, diabetes is associated with increased NET formation, which is linked to slower wound healing [56]. Our data indicate that overproduction of HMGB1 and upregulation of IL-6 promote the formation of NETs, exacerbating HFpEF in T2DM patients. We also show that myocardial Fpassive is significantly elevated in the HFpEF-DM group due to increased inflammation, which correlates with greater symptom severity. Notably, Fpassive was restored with IL-6 inhibition, offering a promising therapeutic strategy for HFpEF-DM.

### Dysregulated signaling pathways contributing to diabetic HFpEF progression

Impaired myocardial insulin signaling leads to excessive free fatty acid (FFA) consumption, which is associated with ROS overproduction [57]. Under these conditions, the heart fails to reduce ROS, decreasing NO bioavailability and endothelial dysfunction [58]. Endothelial dysfunction worsens outcomes in HFpEF patients, contributing to increased cardiomyocyte stiffness and hypertrophy [39]. Our study confirms that NO bioavailability is significantly reduced in HFpEF-DM patients, likely due to post-translational modifications or altered cofactor availability affecting eNOS function.

Furthermore, F_passive_ was significantly increased in HFpEF-DM, correlating with insulin resistance, impaired mitochondrial function, and oxidative stress. However, treatment with mito-TEMPO effectively restored F_passive_ in both groups, consistent with findings from Ni et al., who demonstrated the therapeutic potential of mito-TEMPO in reducing diabetic cardiomyopathy via mitochondrial ROS inhibition [59].

AMPK signaling plays a pivotal role in protecting cardiomyocytes during metabolic disorders like diabetes and obesity. Under metabolic stress, AMPK activity declines, exacerbating cardiac dysfunction. However, AMPK activation mitigates metabolic stress, maintains mitochondrial homeostasis, and stimulates cardiac autophagy [60]. Our study showed elevated p-AMPK in HFpEF-DM, potentially due to anti-diabetic medication effects targeting AMPK phosphorylation, balancing autophagy and cell survival. Metformin administration has been shown to increase AMPK phosphorylation, enhancing eNOS activity and NO bioavailability [61].

Additionally, the interplay between Insulin/PI3K/AKT and AMPK signaling regulates mTOR activity. Insulin-activated PI3K/AKT signaling phosphorylates mTOR, regulating autophagy and cell proliferation [62]. Conversely, AMPK suppresses mTOR during metabolic stress [63]. Our study found that the p-AKT/AKT ratio was significantly lower in HFpEF-DM, consistent with reports linking diminished AKT activation to worsening cardiomyopathy [64]. Additionally, mTOR phosphorylation was downregulated in HFpEF-DM, likely due to anti-diabetic drug-mediated AMPK/mTOR targeting, restoring cardiac function. Although most HFpEF patients in our cohort were receiving statin therapy, which is known to modulate PI3K/AKT/eNOS signaling, the uniform use of statins across both diabetic and non-diabetic groups minimizes the likelihood of a confounding effect on our findings.

### HSP dysregulation and cardiac dysfunction in diabetic hearts

Under physiological conditions, HSPs facilitate protein folding and cardioprotection. However, in failing hearts, inadequate PQC is reflected by decreased HSP levels [65]. Overexpression of HSP70 enhances protein stability, reduces apoptosis, and improves cardiac function under ischemic stress [66]. Shepherd et al. demonstrated that HSP70 expression is reduced in T2DM human hearts, suggesting increased misfolded protein accumulation and ER stress [67].

In agreement with these findings, our study demonstrated a significant reduction in HSP27 and HSP70 expression in HFpEF-DM. This decrease may be attributed to oxidative stress-induced HSP modification, leading to loss of cardioprotective function. Notably, administration of HSP27 and HSP70 restored F_passive_ in HFpEF-DM, aligning with our previous study on αB-crystallin restoring elevated F_passive_ in hypertrophic cardiomyopathy (HCM) [15].

## Conclusion

PQC systems function as an integrated stress response, interacting at both the protein and transcriptional levels. Adequate PQC regulation is critical for diabetic hearts, as imbalanced PQC contributes to HFpEF development via oxidative stress, proteotoxicity, and apoptosis. Targeting PQC mechanisms at different levels could offer a novel therapeutic strategy for preventing HFpEF progression in diabetes, warranting further investigation.

## Data Availability

No datasets were generated or analysed during the current study.

## Abbreviations

T2DM: Type 2 diabetes mellitus
CVD: Cardiovascular disease
CAD: Coronary artery disease
HFrEF: Heart failure with reduced ejection fraction
HFpEF: Heart failure with preserved ejection fraction
FAs: Fatty acids
ROS: Reactive oxygen species
NO: Nitric oxide
PQC: Protein quality control
UPRER: Unfolded protein response in the endoplasmic reticulum
HSPs: Heat shock proteins
HSP70: Heat shock protein70
HSP27: Heat shock proteins27
αB-crystallin: Alpha B-crystallin
AMPK: AMP-activated protein kinase:
mTOR: Mammalian target of rapamycin
NF-κB: Nuclear factor kappa-light-chain-enhancer of activated B-cells
IL-6: Interleukin 6
TNF-α: Tumor necrosis factor
UPS: Ubiquitin–proteasome system
LV: Left ventricle
HMGB1: High mobility group box 1
TLR2: Toll-like receptor 2
TLR4: Toll-like receptor
RAGE: Receptor for advanced glycation end-products
ICAM-1: Intercellular adhesion molecule-1
VCAM-1: Vascular cell adhesion molecule-1
NLRP3: NOD-like receptor protein-3
NET: Neutrophil extracellular traps
PI3K: Phosphoinositide 3-kinase
AKT: Protein kinase B
GSK3B: Glycogen synthase kinase-3 beta
HCM: Hypertrophic cardiomyopathy
IL-6R: Interleukin-6 receptor
ERK 1/2: Extracellular signal-regulated kinases 1/2
MAPK: Mitogen-activated protein kinase
STK11: Serine/threonine kinase 11
NE: Neutrophil elastase
MPO: Myeloperoxidase
eNOS: Endothelial nitric oxide synthase
NOS3: Nitric oxide synthase 3 (endothelial NOS)
sGC: Soluble guanylate cyclase
cGMP: Cyclic guanosine monophosphate
PKG: Protein kinase G
PRKG1: Protein kinase, cGMP-dependent type 1
LPO: Lipid peroxidation
H_2_O_2_: Hydrogen peroxide
qRT-PCR: Quantitative real-time reverse-transcription PCR
